# Depletion of Neonatal Neutrophilic Cells Worsens the Outcome of *E. coli* Sepsis in Newborn Mice

**DOI:** 10.1002/eji.70171

**Published:** 2026-03-29

**Authors:** Julian Schwarz, Janine Hebel, Stefanie Dietz‐Ziegler, Jessica Rühle, Trim Lajqi, Thorsten Orlikowsky, Christian F. Poets, Christian Gille, Natascha Köstlin‐Gille

**Affiliations:** ^1^ Tuebingen University Children's Hospital Department of Neonatology Tuebingen Germany; ^2^ Heidelberg University Children's Hospital Department of Neonatology Heidelberg Germany; ^3^ Aachen University Children's Hospital Department of Neonatology Aachen Germany

**Keywords:** *E. coli*, inflammation, innate immunity, neonatal neutrophilic cells, newborn sepsis

## Abstract

Neonates are particularly susceptible to infections because of a reduced ability to fight bacterial pathogens while simultaneously exhibiting enhanced inflammatory damage. Neonatal neutrophilic cells (NNC) are a heterogeneous population of innate immune cells, which differ substantially from adults. They display functional deficits in pro‐inflammatory responses and include a suppressive subpopulation known as myeloid‐derived suppressor cells (MDSC). It remains unclear whether pro‐ or anti‐inflammatory properties of NNC predominate immediately after birth. This study aimed to clarify the role of neutrophilic cells in neonatal murine sepsis. We established a neonatal mouse model of *E. coli*‐sepsis and depleted NNC using an anti‐Ly6G antibody to assess mortality, bacterial load, cytokine responses, and immune cell composition. A dose of 30,000 CFU *E. coli* resulted in a 67% survival rate in neonatal mice with litters of 5–6 pups, while mortality increased in larger litters. NNC‐depletion significantly increased mortality and systemic inflammation during neonatal sepsis, whereas bacterial load was only minimally affected. Other immune cell populations remained unchanged. *E. coli* sepsis induced pronounced neutrophil infiltration into organs of adults, whereas neonates exhibit reduced numbers of NNC in affected organs. Overall, our findings suggest that NNC‐mediated control of inflammation may protect neonates from lethal sepsis.

## Graphical abstract

In a neonatal mouse model of *E. coli* sepsis, depleting neonatal neutrophilic cells (NNC) increased mortality and systemic inflammation. The bacterial load increased slightly, and the immune cell populations remained unchanged. These findings suggest that NNC may play a role in controlling inflammation and protecting neonates from lethal sepsis.

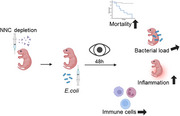

**Abbreviations**
ANC: adult neutrophilic cells
CFSE: carboxyfluorescein‐succinimidyl ester
CFU: colony forming units
DHR: dihydrorhodamine 123
ELISA: enzyme‐linked immunosorbent assay
GR‐MDSC: granulocytic myeloid‐derived suppressor cells
IL‐6: inflammatory cytokine Interleukin 6
IPTG: isopropyl‐β‐D‐thiogalactopyranosid
MACS: magnetic activated cell sorting
MCP‐1: monocyte chemoattractant protein‐1
MDSC: myeloid‐derived suppressor cells
MOI: multiplicity of infection
NNC: neonatal neutrophilic cells
PMA: phorbol‐12‐myristat‐13‐acetat

## 1 Introduction

Neonatal sepsis is one of the most important causes of morbidity and mortality during the neonatal period. The incidence of neonatal sepsis is approximately 0.05% in term‐born infants, but rises to up to 35% in preterm infants [1, 2]. The two most relevant pathogens of neonatal sepsis are group B *streptococci* and *Escherichia coli* [3, 4, 5]. Neutrophils are the first‐line circulating effector immune cells responding to and defending against infections. However, there are significant differences in neutrophil immune responses between adults and neonates [6]. Besides a markedly reduced cell precursor pool, neonatal neutrophils—especially those from preterm infants—exhibit various functional deficits compared to adult neutrophils. Deficits comprise reduced chemotaxis, cell adhesion, and transmigration, as well as diminished production of antimicrobial proteins and reduced phagocytosis [7, 8, 9, 10]. Furthermore, neonatal white blood cell harbors high numbers of neutrophilic cells with immune‐suppressive characteristics, that is, granulocytic myeloid‐derived suppressor cells (GR‐MDSC) [11, 12, 13].

GR‐MDSC are myeloid cells that expand under various pathological conditions such as malignant diseases, trauma, as well as infections, usually leading to harmful immunosuppression especially by targeting T cells. In addition, GR‐MDSC accumulate during pregnancy in the maternal and fetal organism under physiological conditions [11, 14, 15]. While it is evident that GR‐MDSC are crucial for the induction and maintenance of immune tolerance between mother and fetus during pregnancy, their role for immune regulation in the newborn is unclear. On the one hand, they suppress neonatal immune responses such as phagocytosis, antigen presentation, and cytokine production and affect the T‐cell balance in favor of anti‐inflammatory Th2 responses resulting in an increased susceptibility to infections [12, 16, 17, 18, 19, 20]. On the other hand, excessive immune responses in the context of microbiome establishment are suppressed by GR‐MDSC, so that they, for example, protect against necrotizing enterocolitis—a severe inflammatory disease of the premature infant's intestine [21, 22].

One difficulty in investigating the role of GR‐MDSC in vivo is that they are phenotypically indistinguishable from neutrophils. In humans, they express surface markers of myeloid and neutrophil cells such as CD33, CD15, CD11b, and CD66b, but are negative for the monocyte marker CD14 and the MHC‐II molecule HLA‐DR. They differ from neutrophils only in their lower density, as they sediment with mononuclear cells. However, the decisive characteristic distinguishing them from neutrophils is their suppressive activity in vitro. In mice, GR‐MDSC are phenotypically characterized by the expression of CD11b and a high expression of the GR‐1 epitope Ly6G, in contrast to the GR‐1 epitope Ly6C being only weakly expressed (CD11b^+^/Ly6C^lo^Ly6G^+^) [23, 24]. Our group recently showed that Ly6G‐expressing neutrophilic cells isolated from newborn mice had a suppressive effect on T cells in vitro [25].

The role of the suppressive properties of neonatal neutrophilic cells (NNC) in the outcome of neonatal sepsis is yet unclear. In the present study, we aimed to investigate the role of NNC in the pathogenesis of neonatal sepsis in vivo and asked whether they reduce the effectiveness of the bacterial defense or protect against hyperinflammation [20]. For this purpose, we first established a murine neonatal sepsis model based on the subcutaneous inoculation with *E. coli*. Subsequently, we depleted Ly6G‐expressing neutrophilic cells via a depleting antibody. Our results demonstrate that the depletion of NNC increased the mortality of newborn mice during sepsis. Interestingly, the bacterial load in the animals after Ly6G‐depletion was only slightly increased compared to control animals, whereas Ly6G‐depleted animals showed a significantly stronger inflammatory response, as measured by the pro‐inflammatory cytokine interleukin 6 (IL‐6) and monocyte chemoattractant protein‐1 (MCP‐1).

Even though the selective depletion of GR‐MDSC is currently not manageable without the concomitant elimination of mature neutrophils, our data strongly suggest that neutrophilic cells in the newborn exhibit a pronounced suppressive phenotype and thus fulfill the criteria of GR‐MDSC. In our model, NNC appear to improve the course of sepsis and suppress infection‐induced inflammation. Targeting NNC‐functions in neonates might be a promising therapeutic approach to the treatment of neonatal sepsis.

## 2 Methods

### 2.1 Mice

All animals were maintained under pathogen‐free conditions in the research animal facility of Tuebingen University. All experimental animal procedures were conducted according to German federal and state regulations and approved by the regional council Tuebingen (approval number K07/18). Adult non‐pregnant and pregnant C57BL/6J (WT) mice were obtained from Charles River (Sulzfeld, Germany). Neonatal mice used were 1–8 days old. Adult mice used were 8 ‐ to 12‐week‐old. Male and female mice were used.

### 2.2 Neonatal Sepsis Induction

*E. coli*, an encapsulated K1 wildtype strain from a clinical meningitis was a kind gift of Dr. Matthias Marschal, University of Tuebingen, Germany.

Bacteria were pre‐cultured on agar plates and then adjusted in PBS to 10,000, 30,000, or 100,000 colony forming units (CFU) in 30 µL PBS. Sepsis was induced in newborn mice either on postnatal Day 2 (P2) or Day 8 (P8) by subcutaneous injection of *E. coli* K1 in different doses (10,000 CFU/mouse, 30,000 CFU/mouse, 100,000 CFU/mouse, or 20,000 CFU/g bodyweight). For the comparison with the adult sepsis was induced on P2 with 30,000 CFU/mouse. Injection of PBS was used as a control. After sepsis induction, mice were monitored for signs of sepsis using existing scoring systems [26, 27, 28] with modifications. The scoring system comprised assessment of respiration, mobility, general appearance, alertness, appearance of the injection site, and maternal behaviour. In addition, mice were weighed at the time of sepsis induction and then every 12 h during the experiment. The experiment was terminated when defined termination criteria (decreasing mobility, level of consciousness or respiration quality, rejection by the mother and swelling of the injection site) were reached or 48 h after induction of sepsis. For the establishment of the neonatal sepsis model, animals from litters of varying sizes (5–9 pups) were used. For the final experiments with NNC depletion, only animals from litters that naturally contained 5–6 pups were included.

### 2.3 Adult Sepsis Induction

For the adult sepsis induction, the same *E. coli* described above were used. Around 8‐ to 12‐week‐old mice were separated into individual cages 1 day prior to the start of the experiment to reduce stress on the day of the experiment. For sepsis induction, the mice received an intraperitoneal (i.p.) injection of 6 × 10^7^ CFU *E. coli* in 200 µL PBS. Injection of PBS was used as a control. After sepsis induction, mice were closely monitored for signs of sepsis using a scoresheet and a point system, approved by the regional council in Tübingen. The scoring system comprised loss of appetite and severe weight loss, reduced activity, piloerection (raised fur) on the back and bridge of the nose, mucus around the eyes, a hunched posture, and reduced or no reaction to stimuli. The experiment was terminated when defined termination criteria were reached or 96 h after induction of sepsis.

### 2.4 Depletion of Ly6G‐Expressing Cells

To study the effect of NNC depletion on the course of neonatal sepsis, newborn mice were administered either an intraperitoneal injection of 10 µg/mouse of anti‐Ly6G (1A8, anti‐mouse Ly6G, Bio X Cell Lebanon, USA) to deplete Ly6G‐expressing cells, or 10 µg/mouse of isotype control antibody (2A3, rat IgG2a isotype control, Bio X Cell Lebanon, USA) 1 day prior to sepsis induction. The safety and feasibility was shown, for example, by Pocratsky et al. [29].

### 2.5 Tissue Collection and Single‐Cell Preparation

Blood, spleens, livers, lungs, and peritoneal fluid from newborn mice were collected 48 h after sepsis induction or when termination criteria were reached. Spleens, livers, and lungs from adult mice were collected 96 h after sepsis induction or when termination criteria were reached. Spleens from adult mice were used for isolation of CD4^+^ T cells for T‐cell proliferation assays. In addition, spleens from neonatal (P2) and adult mice were obtained for the functional investigations of NNC and adult neutrophilic cells (ANC). To obtain single cell suspensions, tissue was pushed through a 40 µm filter (Greiner bio‐one, Frickenhausen, Germany) using a syringe plunger. Single cell suspensions were adjusted to 1–4 × 10^6^ cells/mL in PBS.

### 2.6 Quantification of Bacterial Load

For quantification of the bacterial load in organs after sepsis induction, organs were weighed and then pushed through 40 µm filters (Greiner bio‐one, Frickenhausen, Germany) with 2 mL PBS. Blood (30–200 µL) and peritoneal lavage (100–200 µL) were diluted with 1 mL PBS. Different dilutions ranging from 1:10 to 1:10,000,000 were plated on Columbia agar plates supplemented with 5% sheep blood (Thermo Fisher Scientific, Wesel, Germany) and cultured for 24 h at 37°C with 5% CO_2_. CFU were counted, and CFU per gram organ weight or per mL of fluid was determined.

### 2.7 Cell Isolation

For the isolation of Ly6G expressing adult and neonatal NC from murine splenocytes by magnetic activated cell sorting (MACS), splenocytes were labeled with Gr‐1 biotin‐antibody and then separated using Streptavidin microbeads. This was followed by a second isolation step using Ly6G biotin‐antibody and anti‐biotin microbeads (modified protocol of MDSC Isolation Kit mouse, Miltenyi Biotec, Bergisch‐Gladbach, Germany). The purity of the adult and neonatal NC fraction after separation was determined by flow cytometry. Only fractions with a purity >90% were used for subsequent analysis.

For isolation of CD4^+^ T cells from adult splenocytes, we followed the manufacturer's instructions using a T‐cell Biotin‐antibody cocktail followed by two sequential anti‐biotin magnetic bead separation steps (Miltenyi Biotec, Bergisch‐Gladbach, Germany). The purity of CD4^+^ T cells after separation was greater than 90%, assessed by flow cytometry.

### 2.8 Flow Cytometry

For extracellular staining, freshly isolated cells were washed in FACS buffer before fluorescent‐conjugated extracellular antibodies were added. Antibodies were purchased from BD Biosciences (CD3 FITC [145‐2C11], CD4 APC [RM4‐5], CD11b FITC [M1/70], CD45 PerCp [30‐F11], TLR4 [CD284/MD‐2] PE [MTS510] FSV510 [Amcyan], from BioLegend CD4 APC‐Cy7 [GK1.5], CD19 PE [1D3], CD25 APC [PC61] Gr‐1 PE‐Cy7 [RB6‐8C5], Ly6G APC [1A8], NKp46 PE‐Cy7 [29A1.4], CD11c BV421 [N418], F4/80 APC [BM8] MHC II APC‐Cy7 [M5/114.15.2], 7‐amino‐actinomycin D [7AAD], Annexin V [PE/Cyanine7] or Miltenyi Biotec Ly‐6G FITC [REA526]).

For immune cell quantification, cell‐doublets were excluded and cells were pre‐gated for living CD45^+^ cells. Among these, immune cell subset from spleens and livers were identified as followed: T cells by CD3^+^, activated T cells by CD3^+^/CD4^+^/CD25^+^, B cells by CD3^−^/CD19^+^, NK‐cells by CD3^−^/NKp46^+^, NNC and ANC by CD11b^+^/ Gr‐1^+^ (in Figure 2), or by CD11b^+^/ Ly‐6G ^+^(in all other figures), dendritic cells by CD11c^+^/MHCII^+^, macrophages by CD11c^−^/F4/80^+^, monocytes by CD11b^+^/Gr‐1^−^. The gating strategy is shown in Figure S5.

For detection of intracellular cytokines, spleen cells from newborn mice were cultured overnight and then stimulated for 4 h with *E. coli* (inactivated, MOI 1:50) and brefeldin A (BD Bioscience). After that, cells were harvested and extracellular staining was performed. Cells were washed with FACS buffer, 100 µL Cytofix/Cytoperm (BD Bioscience) was added, and cells were incubated for 30 min at 4°C. Cells were then washed with Perm/Wash buffer (BD Pharmingen) and intracellular antibody for IL‐6 APC (MP5‐20F3, BioLegend), MCP‐1 PE (2H5, BioLegend), IL‐1β APC (REA577, Miltenyi), and TNF‐α PE (MP6‐XT22, BD Bioscience), (all BD Bioscience) were added. After a 30 min incubation, cells were analyzed.

Data acquisition was performed with a FACS Canto II flow cytometer (BD Bioscience) and analyzed with FlowJo V10 (FlowJo, LLC, Ashland, Oregon, USA)

### 2.9 ROS Measurement

For detection of ROS, 5 × 105 spleen cells were incubated with dihydrorhodamine 123 (DHR, Sigma‐Aldrich, Munich, Germany, c end: 100 mM) in RPMI containing Ca2+ and Mg2+ for 5 min at 37°C. After that, cells were stimulated for 15 min with Phorbol‐12‐myristat‐13‐acetat (PMA, Sigma‐Aldrich, Munich, Germany, c end: 1 µM). Surface staining with CD11b and Ly6‐G was performed, and ROS production was analyzed by flow cytometry.

### 2.10 T‐Cell Suppression Assay

Freshly isolated CD4^+^ T cells isolated from splenocytes of adult mice were stained with carboxyfluorescein‐succinimidyl ester (CFSE, Invitrogen, Carlsbad, USA) following the manufacturer´s instructions. CFSE‐labeled CD4^+^ T cells were stimulated with mouse T‐activator CD3/CD28 Dynabeads (Thermo Fisher Scientific, Waltham, USA) and 50 ng recombinant murine interleukin‐2 (rmIL‐2, R&D Systems, Minneapolis, USA) in the presence of ß‐mercaptoethanol (Merck, Darmstadt, Germany) at a concentration of 50 mM. NC isolated from spleens of newborn mice at P2 or adult mice were also suspended in media and added in different ratios to CFSE‐stained and stimulated T cells (neonatal: NNC:T cells 1:2, 1:4, 1:8, and 1:16; adult: ANC:T cells 1:2). After 4 days of culture, CD4^+^ T‐cell proliferation was determined by flow cytometry using CFSE dye dilution. The proliferation index, defined as the ratio of CD4^+^ T‐cell proliferation after addition of NNC/ANC versus CD4^+^ T‐cell proliferation without NNC/ANC was determined. CD4^+^ T‐cell proliferation without NNC/ANC was set to a fixed value of 1. In addition, after 4 days of culture the viability the NNC was measured using a AnnexinV and 7‐AAD Apoptosis Detection Kit I (BioLegend) according to the manufacturer's instructions and analyzed by flow cytometry.

### 2.11 Phagocytosis Assay

*E. coli* expressing GFP was cultured in LB medium supplemented with 50 µL/mL Isopropyl‐β‐D‐thiogalactopyranosid (IPTG) and 50 µg/mL kanamycin. The culture was incubated overnight at 37°C with shaking at 200 rpm. The following day, 200 µL of the overnight bacterial culture was transferred to fresh medium, and IPTG and kanamycin were added as before. The culture was then incubated for 1 h at 37°C. Incubation with splenocytes was performed for 60 min at a multiplicity of infection (MOI) of 50:1. The cells were analyzed by flow cytometry.

### 2.12 Measurement of Protein Concentration and Cytokine Production

For the quantification of serum cytokine concentration, initially total protein concentration in sera of newborn animals was quantified using the Pierce 660 nm Protein Assay Kit (#22662) from Thermo Fisher Scientific (Massachusetts, USA) as described [30, 31]. Briefly, 10 µL of protein standard, diluted samples, and blank were plated in a 96‐well plate, followed by the immediate addition of 150 µL of assay reagent. Subsequently, the plate was covered and the solutions mixed for 1 min using a plate shaker. Afterwards, the plate was incubated for an additional 5 min at room temperature without shaking. Absorbance was measured at 660 nm using an iMark Microplate Reader (Bio‐Rad Laboratories, Hercules, CA, USA). Protein concentrations were then calculated based on the values of the standard curve.

After termination of the experiment or 48 h after beginning of the experiment if the pups survived IL‐6 (#431302) and MCP‐1 (#432701) concentration, in serum samples collected, was measured using commercial enzyme‐linked immunosorbent assay (ELISA) kit obtained from BioLegend (San Diego, CA) as described [31, 32]. Briefly, blood samples were collected and centrifuged for 10 min at room temperature at 400 × *g* and stored at −80°C until further processing. The ELISA kit was used according to the manufacturer´s instructions. The test absorbance was measured at 450 nm with a reference wavelength at 570 nm employing an iMark Microplate Reader (Bio‐Rad Laboratories, Hercules, CA, USA). IL‐6 and MCP‐1 levels were normalized to the total protein concentrations of each sample.

### 2.13 Statistical Analysis

Statistical analysis was performed using GraphPad Prism 10.1.1 (GraphPad Software, La Jolla, CA). Data were analyzed for Gaussian distribution using the Shapiro–Wilk test comparisons between more than two groups of unpaired and not normally distributed data were analyzed using the Kruskal–Wallis test. Comparisons between more than two groups of unpaired and normally distributed data were analyzed using a one way ANOVA. Comparisons between two groups of unpaired and not normally distributed data were evaluated using the Mann–Whitney test. Comparisons between two groups of unpaired and normally distributed data were evaluated using the unpaired *t* test. A *p*‐value <0.05 was considered as statistically significant.

Survival rates were determined based on Kaplan–Meier survival analysis by comparing the survival of different dosages using the log‐rank (Mantel–Cox) test.

## 3 Results

### 3.1 Establishment of a Neonatal Sepsis Model With *E. coli* in Mice

**FIGURE 1:**
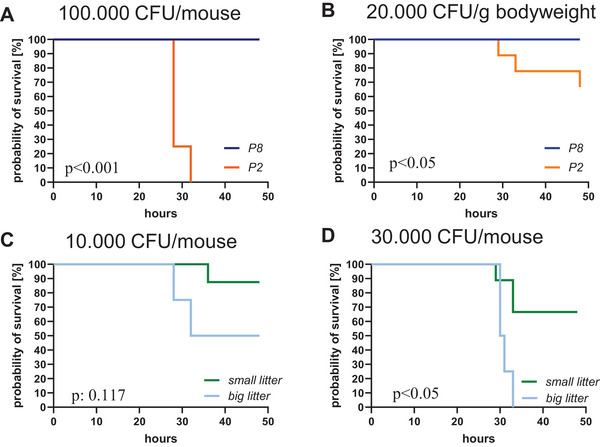
Sepsis mortality of newborn mice. (A–D) Sepsis was induced in newborn mice either at the second or eighth day after birth (P2 or P8) by subcutaneous injection of 100,000 CFU, 30,000 CFU, or 10,000 CFU *E. coli* per mouse in total or 20,000 CFU *E. coli* per gram bodyweight. Pups were thoroughly monitored and sacrificed when reaching the criteria for critical illness or 48 h after sepsis induction. (A) Probability of survival after subcutaneous injection of 100,000 CFU *E. coli* as a function of the postnatal day of sepsis induction. P2 *n* = 4, P8 n = 6; *p* < 0.001, log‐rank (Mantel–Cox) test. (B) Probability of survival after subcutaneous injection of 20,000 CFU *E. coli* per gram bodyweight as a function of the postnatal day of sepsis induction. P2 *n* = 9; P8 *n* = 6; *p* < 0.05, log‐rank (Mantel–Cox) test. (C) Probability of survival after subcutaneous injection of 10,000 CFU *E. coli* at P2 as a function of the litter size. Small litter *n* = 8, big litter *n* = 4; *p* < 0.117, log‐rank (Mantel–Cox) test. (D) Probability of survival after subcutaneous injection of 30,000 CFU *E. coli* at P2 as a function of the litter size. Small litter *n* = 9, big litter *n* = 4; *p* < 0.05, Log‐rank (Mantel–Cox) test. Each *n* is representing a single mouse Mice for the experiments came from two to four litters (A–D).

To investigate the role of neutrophilic cells in the pathogenesis of neonatal sepsis, we first established a neonatal *E. coli* sepsis model aiming for a survival rate of approximately 50%. We first tested two different time points for sepsis induction and different bacterial dosages. For this purpose, we induced sepsis with 100,000 CFU of *E. coli* in newborn mice either early after birth (on postnatal Day 2, P2) or in the late neonatal period (on postnatal Day 8, P8). Early sepsis induction at P2 resulted in a 0% survival rate in pups (*n* = 4), while after late induction at P8 all pups (*n* = 6) survived for 48 h (*p* < 0.001, Figure 1A). Adjusting the dosage to the body weight to 20,000 CFU/g resulted in a survival rate of 66% in pups at P2 (*n* = 9) and 100% at P8 (*n* = 6) after 48 h (*p* < 0.05, Figure 1B). Notably, litter size had a significant impact on survival after sepsis induction on P2, with lower survival observed in mice raised in larger litters (8–9 pups) compared to those raised in smaller litters (5–6 pups). When an absolute dosage of 10,000 CFU *E. coli* per mouse was used, 50% of the pups of large litters (*n* = 4) and 88% of the small litter pups (*n* = 8) survived (not significant, Figure 1C). After sepsis induction with 30,000 CFU per mouse, no pups of the large litter (*n* = 4) and 67% of the small litter (*n* = 9) survived (*p* < 0.05, Figure 1D). Interestingly, pups from large litters had significantly higher body weights compared to those from small litters (Figure S1A,B). Based on our observations, we used newborn mice at P2 from litters with 5–6 animals for further experiments. Figure S1C summarizes the survival rates after different doses of *E. coli* in mice on P2 in small litters.

### 3.2 Neonatal Sepsis Leads to Pronounced Neutropenia

**FIGURE 2:**
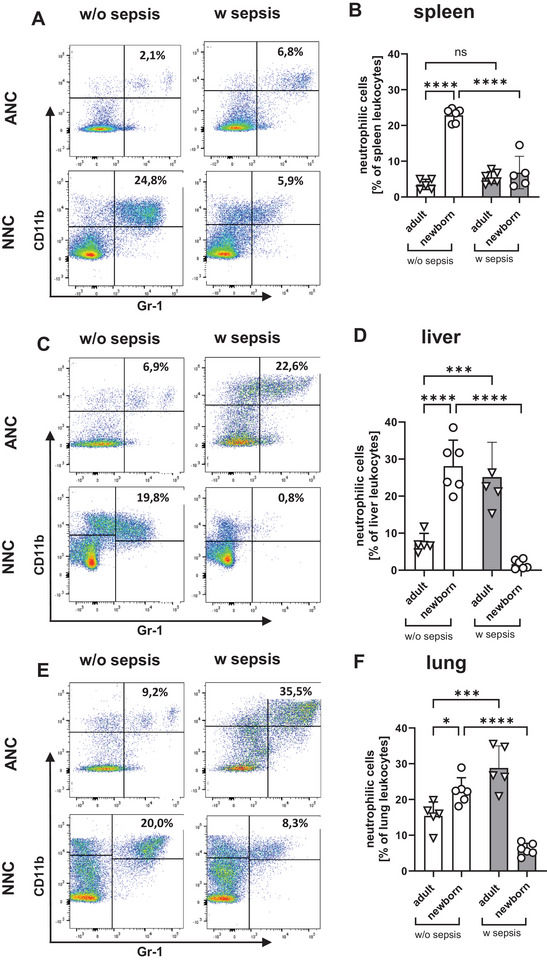
Organ influx of neutrophilic cells during adult and neonatal *E. coli* sepsis. Sepsis was induced in newborn mice (P2) and adult mice by subcutaneous injection of *E. coli* (P2: 30,000 CFU/mouse, adult: 6 × 10^7^ CFU/mouse). Injection of PBS was used as control. The mice were thoroughly monitored and sacrificed when reaching the criteria for critical illness or 48 h (neonatal) or 96 h (adult) after sepsis induction. Spleens (A+B), livers (C+D), and lungs (E+F) were collected and homogenized to obtain single cell suspensions which were analyzed by flow cytometry. (A, C, E) Representative density plots showing neutrophilic cells in organs of adult (upper row, ANC) and neonatal (lower row, NNC) mice without sepsis (left row, w/o sepsis) or with sepsis (right row, w sepsis). Cells were pre‐gated for living CD45^+^ (B, D, F) Bar graphs showing percentages of ANC and NNC in organs of adult and newborn mice without sepsis (white bars, w/o sepsis) or with sepsis (grey bars, w sepsis). Bars show mean and standard deviation from 5 to 6 independent experiments, ns = not significant, *p < 0.05, ***p < 0.001, ***p < 0.001, ****p < 0.0001; one way ANOVA. Each dot (= *n*) represents an individual mouse. Mice for the experiments came from two litters for the experiments with NNC (A–F).

We next analyzed numbers of neutrophilic cells in spleens, livers, and lungs of newborn and adult mice without and with *E. coli* sepsis. Neutrophilic cells were defined as CD11b^+^/Gr‐1^+^ leukocytes. Compared to adult mice, neonatal mice without sepsis exhibited significantly higher proportions of neutrophilic cells in the analyzed organs spleen (22.8% vs. 3.5% *p* = 5–6, ****p < 0.0001) liver (28.1% vs. 7.4% *p* = 5–6, ****p < 0.0001), and lung (22.4% vs. 15.1% *p* = 5–6, *p < 0.05). Whereas sepsis in adult mice led to neutrophil infiltration in the liver (7.4% vs. 25.1% *p* = 5–6, ***p < 0.001) and lung (15.4% vs. 28.8% *p* = 5–6, ***p < 0.001), septic neonatal mice showed a marked reduction of neutrophilic cells across all organs (spleen: 22.8% vs. 6.8% *p* = 5–6, ****p < 0.0001; liver: 28.1% vs. 1,6% *p* = 5–6, ****p < 0.0001; lungs: 22.4% vs. 6.1% *p* = 5–6, ****p < 0.0001) (Figures 2A–F).

### 3.3 NNC and ANC Differ in Their Functional Properties

**FIGURE 3:**
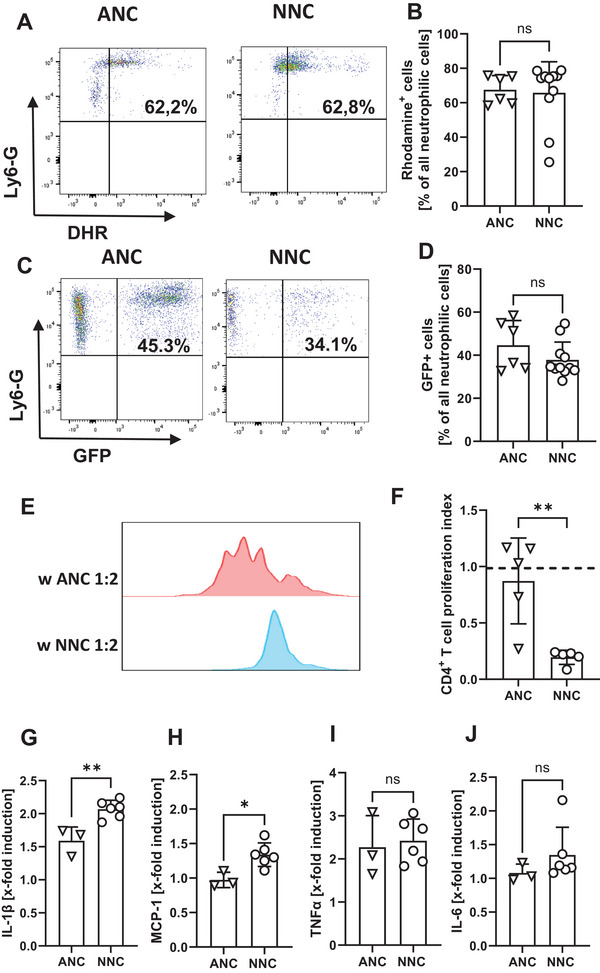
In vitro functional activity of adult and neonatal neutrophilic cells. Newborn mice at the first day of life (P1) and adult mice were euthanized, spleens were collected and homogenized to obtain single cell suspensions. (A+B) Cells were then incubated with dihydrorhodamine 123 (DHR) and stimulated for 15 min with PMA. ROS production of neutrophilic cells was analyzed by measuring Rhodamine expression by flow cytometry. (A) Representative density plots showing percentages of Rhodamine^+^ neutrophilic cells (upper right quadrant) in ANC (left) and NNC (right). (B) Bar graphs showing percentages of Rhodamine^+^ cells in splenic ANC and NNC. Bars show mean and standard deviation from 6 to 11 independent experiments, ns not significant, Mann–Whitney test. (C+D) Cells were incubated with GFP‐expressing *E. coli* for 1 h at a MOI of 50:1. Phagocytosis capacity was measured by quantifying GFP‐expressing cells by flow cytometry. (C) Representative density plots showing the phagocytosis rate of ANC (left) and NNC (right). (D) Bar graphs showing percentages of GFP^+^ cells in splenic ANC and NNC. Bars show mean and standard deviation from 6 to 11 independent experiments, ns not significant, Mann–Whitney test. (E+F) ANC and NNC were isolated by MACS and added to CD4^+^ T‐cells, freshly isolated from spleens of adult mice, stained with CFSE and stimulated with anti‐CD3/CD28 microbeads in a 1: 2 ratio. After 4 days, proliferation of CD4^+^ T‐cells was assessed by CFSE dye dilution. The proliferation index was determined as ratio of T‐cell proliferation with and without addition of NNC/ANC. (E) Representative histogram plots showing proliferation of stimulated CD4^+^ T‐cells with the addition of ANC (red histogram) and with the addition of NNC (blue histogram). (F) Bar graph showing the inhibitory effect of NNC and ANC on CD4^+^ T‐cell proliferation. Proliferation of CD4^+^ T‐cells after stimulation and without addition of NNC or ANC was set to 1 (dashed line). Bars show mean and standard deviation from 5 to 6 independent experiments; **p < 0.01; unpaired t test. (G–J) Spleen cells were cultured overnight and then stimulated for 4 h with heat‐inactivated *E. coli* at a MOI of 1:50. Intracellular cytokine expression was analyzed by flow cytometry. (G–J) Bar graphs showing x‐fold induction of IL‐1 β (G), MCP‐1 (H), TNF‐α (I), and IL‐6 (J) of splenic ANC and NNC after *E. coli* stimulation. Bars show mean and standard deviation from 3 to 6 independent experiments, *p < 0.05, **p < 0.01, unpaired *t* test. Each dot (= *n*) represents an individual mouse. Mice for the experiments came from 2 to 4 litters.

Next, we comparatively investigated the functional properties of ANC and NNC. As NNC were depleted via Ly6G in the subsequent sepsis experiments, NNC and ANC were defined as CD11b^+^/Ly6G^+^ cells in the functional assays. While there was no difference between ANC and NNC in ROS production (67.0% vs. 65%, *n* = 6–11, Figure 3A,B) and phagocytosis of *E. coli* between NNC and ANC (44.6% vs. 37.8%, *n* = 6–11 Figure 3C,D), we observed a clear difference in T‐cell suppressive activity of NNC and ANC. ANC had no impact on CD4^+^ T‐cell proliferation, while NNC showed strong suppressive effects (proliferation index 87% for ANC and 19.5% for NNC, *n* = 5–6, **p < 0.01, Figure 3E,F). Figure S2A,B shows concentration dependency of suppressive activity of NNC. Figure S2C,D shows viability of NNC after 4 days of culture. Upon *E. coli* stimulation, NNC exhibited a stronger induction of IL‐1β (1.59 vs. 2.07, *n* = 3–6, **p < 0.01) and MCP‐1 (0.97 vs. 1.33, *n* = 3–6, *p < 0.05) expression compared to ANC. TNF‐α was similarly induced in ANC and NNC while IL‐6 expression remained unchanged in both groups (Figure 3G–J).

### 3.4 Depletion of Ly6G‐Expressing NNC Leads to Increased Sepsis Mortality in Newborn Mice

**FIGURE 4:**
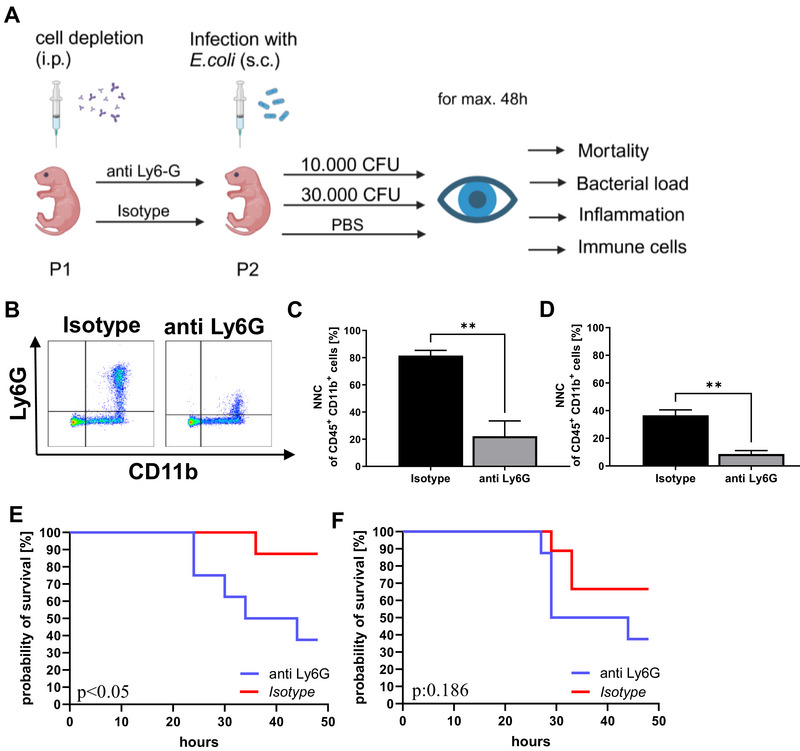
Sepsis mortality after depletion of Ly6G‐positive NNC. (A) Schematic representation of the experimental procedure. (B–D) Newborn mice received either an intraperitoneal injection of an anti‐Ly6G antibody to deplete NNC or an isotype control antibody on the first postnatal day (P1). At P2 or P8 spleen cells were isolated and analyzed by flow cytometry. (B) Representative density plots showing the population of CD11b^+^/Ly6G^+^ (upper right quadrant) NNC in newborn mice at P2 after administering either isotype control (left side, Isotype) or the depleting antibody (right side, anti Ly6G). Cells were pre‐gated for living CD45^+^ cells. (C, D) Bar graphs showing percentages of total NNC from CD45^+^/CD11b^+^ myeloid cells in spleens of newborn mice at P2 (C; 1 day after depletion; isotype: *n* = 7, anti Ly6G: *n* = 5) and P8 (D; 7 days after depletion; isotype: *n* = 5, anti Ly6G: *n* = 5). Bars show mean and standard deviation, **p < 0.01, Mann–Whitney test. (E, F) Newborn mice received either an intraperitoneal injection of an anti‐Ly6G antibody to deplete NNC or an isotype control antibody at the first day of life (P1). At P2, mice were injected subcutaneously with either 10,000 CFU or 30,000 CFU *E. coli*. The pups were sacrificed when reaching the criteria for critical illness, or 48 h after sepsis induction. Probability of survival after subcutaneous injection of 10,000 CFU *E. coli* (E; anti Ly6G *n* = 8, isotype *n* = 8; *p < 0.05, log‐rank [Mantel–Cox] test) or 30,000 CFU *E. coli* (F; anti Ly6G *n* = 8, isotype *n* = 9; p:0,186: not significant, log‐rank [Mantel–Cox] test) in mice after NNC‐depletion (anti Ly6G) or injection of an isotype control (isotype). Each *n* is representing a single mouse. Mice for the experiments came from three litters (B–F).

We now aimed to investigate the role of NNC during the progression of neonatal sepsis. For this purpose, we depleted Ly6G‐expressing NNC in newborn mice on the first postnatal day (P1) by administering a depleting anti‐Ly6G antibody intraperitoneally (Figure 4A). Ly6G‐expressing NNC were significantly diminished in the spleens 1 day after administering the depleting antibody (Figure 4B,C). Surprisingly, even seven days after administration, there was still a significant reduction in Ly6G‐expressing NNC compared to control animals (Figure 4D). The remaining NNC after Ly6G depletion exhibited T‐cell suppressive activity to a much lesser extent (Figure S2E). Depletion of NNC resulted in reduced survival compared to control animals, both when a low dose (10,000 CFU) and a high dose (30,000 CFU) of *E. coli* were applied (38% vs. 88%, *n* = 8, *p* < 0.05, after infection with 10.000 CFU *E. coli* and 38% vs. 66%, *n* = 8, not significant: 0.186, after infection with 30,000 CFU *E. coli*, Figure 4E,F). In addition, weight gain after sepsis induction was slightly lowered in Ly6G‐depleted mice compared to control animals (Figure S3A,B).

### 3.5 Increased Bacterial Load and Increased Inflammation During Neonatal Sepsis After Depletion of Ly6G‐Expressing NNC

**FIGURE 5:**
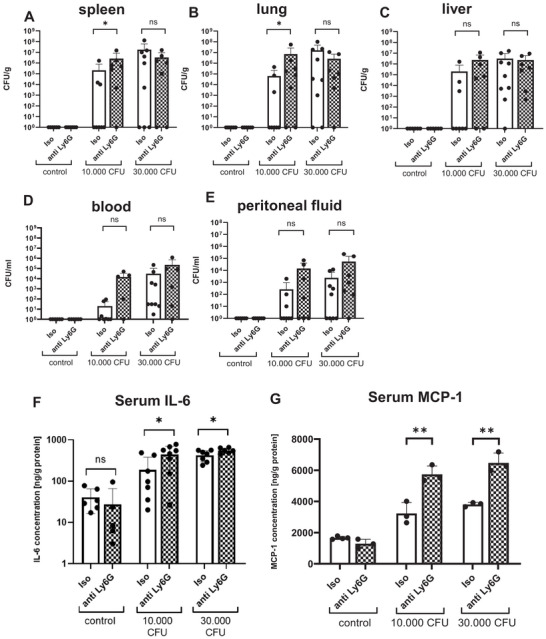
Bacterial load and serum concentrations of IL‐6 and MCP‐1 after depletion of Ly6G‐positive NNC. Newborn mice received either an intraperitoneal injection of an anti‐Ly6G antibody (anti Ly6G) to deplete NNC or an isotype control antibody (Iso) at the first day of life (P1). At P2, mice were injected subcutaneously with either 10,000 CFU or 30,000 CFU *E. coli*. Injection of PBS served as negative control. The pups were sacrificed when reaching the criteria for critical illness or 48 h after sepsis induction. Spleens, lungs, livers, blood, and peritoneal lavage were collected. Organs were homogenized and samples were diluted with PBS. Suspensions were incubated for 24 h on Columbia agar plates with 5% sheep blood and colony forming units (CFU) were counted to calculate CFU per gram (g) organ weight or per milliliter (mL) fluid (blood or peritoneal lavage). Scatter diagrams with bars showing CFUs per gram organ weight (A–C) or per ml (D, E) in spleens (A), livers (B), lungs (C), blood (D), and peritoneal fluid (E) of control mice (Iso, white bars) or NNC‐depleted mice (anti‐Ly6G, striped bars) without sepsis induction (control, left side) or with sepsis induction with 10,000 CFU (middle) or 30,000 CFU (right side). Bars show mean and standard deviation. *n* = 5–9, * *p* < 0.05, ns: not significant, Mann–Whitney test. (F, G) Newborn mice received either an intraperitoneal injection of an anti‐Ly6G antibody (anti Ly6G, checked bar) to deplete NNC or an isotype control (Iso, plain bar) on the first day of life (P1). On P2, newborn mice from a litter with a size of 5–6 pups were injected subcutaneously with either 10,000 CFU (middle, *n* = 5–8) or 30,000 CFU (right side, *n* = 5–8) or with PBS as a negative control (left side, *n* = 6). After termination of the experiment or 48 h after beginning of the experiment, if the pups survived IL‐6 (F) and MCP‐1 concentration in serum samples was measured using enzyme‐linked immunosorbent assay (ELISA) IL‐6 (F) and MCP‐1 (G) concentration in serum samples was measured. The IL‐6 and MCP‐1 levels were normalized to the protein concentrations of each sample. Bars show mean and standard deviation. *n* = 3–8, *p < 0.05, **p < 0.01; unpaired *t* test. Each dot (= *n*) is representing a single mouse. Mice for the experiments came from three litters (A–G).We further investigated systemic inflammation during neonatal sepsis with and without depletion of Ly6G‐expressing NNC by measuring the levels of the proinflammatory cytokines IL‐6 and MCP‐1 in serum of the animals. Both, IL‐6 and MCP‐1 levels were significantly increased after Ly6G‐depletion both after sepsis induction with 10,000 (IL‐6: 187 ng/g vs. 443 ng/g; *n* = 7, 8; *p* < 0.05; MCP‐1: 3231 ng/g vs. 5737 ng/g; *n* = 3; **p < 0.01) or 30.000 CFU *E. coli* (IL‐6: 422 ng/g vs. 552 ng/g, *n* = 3, *p* < 0.05; MCP‐1: 3808 g/g vs. 6470 g/g, *n* = 3; **p < 0.01) (Figure 5F,G).

Spleens and lungs showed an increased bacterial load after Ly6G depletion when using the low (10,000 CFU) *E. coli* dose (2.6 × 10^6^ CFU/g vs. 2.1 × 10^5^ CFU/g for spleens; iso: *n* = 8, anti Ly6G: *n* = 7, *p* < 0.05 and 7.1 × 10^6^ CFU/g vs. 6.4 × 10^4^ CFU/g for lungs; iso: *n* = 8, anti Ly6G: *n* = 7, *p* < 0.05, Figure 5A,B). In all other organs, no significant differences were observed, neither after sepsis induction with the low nor with the high dose (Figure 5C–E). CFU in spleens and blood was only marginally higher in mice who died compared to mice who survived during the sepsis (Figure S4A,B).

### 3.6 Impact of Depletion of Ly6G Expressing NNC on Immune Cell Populations During Neonatal Sepsis

**FIGURE 6:**
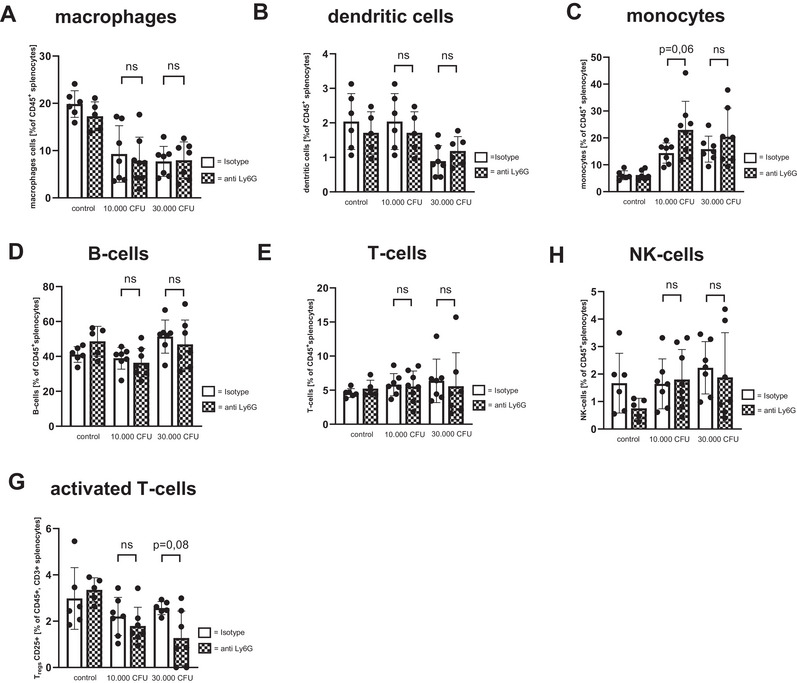
Immune cell populations in spleens after depletion of Ly6G‐positive NNC. Newborn mice received either an intraperitoneal injection of an anti‐Ly6G antibody (anti Ly6G) to deplete NNC or an isotype control antibody (Isotype) at (P1). At P2 mice were injected subcutaneously with either 10,000 CFU or 30,000 CFU *E. coli*. Injection of PBS served as negative control. The pups were sacrificed when reaching the criteria for critical illness or 48 h after sepsis induction. Spleens were collected and homogenized to obtain single cell suspensions. Immune cell subsets were analyzed by flow cytometry. Scatter diagrams with bars showing percentages of macrophages (A), dendritic cells (B), monocytes (C), B cells (D), T cells (E), and NK cells (F) and activated T cells of leukocytes of control mice (isotype, white bars) or NNC‐depleted mice (anti‐Ly6G, striped bars) without sepsis induction (control) or with sepsis induction with 10,000 CFU or 30,000 CFU. *n* = 6–7, unpaired *t* test. Each dot (= *n*) is representing a single mouse. Mice for the experiments came from three litters.

When looking at the immune cell populations we found only marginal impact of depletion of Ly6G‐expressing NNC. Sepsis generally caused a decrease and macrophages (Figure 6A, as well as a less pronounced decrease in dendritic cells (Figure 6B) and a rise in monocytes (Figure 6C) collected from spleens of Ly6G‐depleted mice as well as from control animals. The depletion of NNC led to slightly but not significantly increased levels of monocytes at a dosage of 10,000 CFU. B cells, T cells, and NK cells remained unaffected by sepsis induction (Figure 6D–F) and again, Ly6G depletion had hardly any effect on major lymphocyte populations. The number of CD25^+^ expressing activated T cells were also slightly but not significantly decreased in spleens after sepsis induction with both doses of *E. coli* in Ly6G‐depleted animals (Figure 6G). Figure S5 shows the gating strategy for immune cell quantification. A similar effect was observed in immune cell populations in the livers (Figure S6).

## 4 Discussion

Human neonates and especially preterm infants are much more susceptible to infection than adults, which is attributed to the neonatal type of their immune response [2, 33]. Neutrophils are one of the first line immune cells that respond to upcoming infections. However, there are clear functional differences between adult and NNC with a physiologic expansion of GR‐MDSC during fetal and neonatal life, making NNC being a heterogeneous cell population [7, 8, 9, 10, 34, 35]. In the current study, we investigated the role of NNC during neonatal *E. coli* sepsis and aimed to dissect pro‐ and anti‐inflammatory components of this heterogeneous cell population.

Various murine neonatal sepsis models had been published which used different application routes (intraperitoneal, subcutaneous, intravenously), different ages (P2–P7) and different causative pathogens, such as single pathogens like *E. coli*, GBS, *Listeria monocytogenes* and others, or polymicrobial sepsis models induced by cecal slurry [21, 26, 28, 36, 37, 38, 39, 40, 41, 42, 43]. In our model, we induced sepsis by subcutaneous application of *E. coli* K1 and achieved a mortality of about 35% with a dose of 30,000 CFU in animals at P2. This is similar to the experimental setup of Singh et al. who also used *E. coli* K1 in mice at P2 but used 100,000 CFU to achieve a mortality of 50% [44]—a dosage at which all animals in our setting died. These differences could be due to the different mouse strain used BalbC animals in the study of Singh et al. and C57BL/6J animals in our study. In addition, different environmental conditions such as bacterial cultivation, husbandry, microbiome of the experimental animals, etc. probably also play a role. Interestingly, we observed that the litter size had a significant impact on the survival rate, based on the observation that animals from smaller litters displayed an improved resilience. Contrary to our expectations, this was not an effect of body weight. Individual animals in smaller litters had a lower weight in contrast to those in larger litters. Despite the fact that these individuals were exposed to an increased relative bacterial load, they better resisted the bacterial encounter. Therefore, we assumed that the care and feeding by the mother, which is probably more intense in smaller litters, plays a larger role in the course of sepsis than the initial weight. To our knowledge, the influence of litter size on the course of experimental sepsis has not yet been investigated.

Assessment of numbers of neutrophilic cells in organs of neonatal and adult mice without and with *E. coli* sepsis showed that there is an influx of neutrophilic cells in organs of adult mice, while in newborn mice, NNC decrease sharply. This is in line with previous studies and the fact that immature neutrophils from the bone marrow migrate into the peripheral organs during adult sepsis [45]. In neonates, bone marrow capacity is far more reduced which often results in neutropenia [46].

Functional in vitro experiments comparing NNC with ANC showed that there are no differences in ROS production and phagocytosis of *E. coli*. This is similar to our own previous works in human neonates, where we saw a comparable phagocytosis capacity in neutrophils isolated from cord blood and adult blood [47]. In contrast to NNC, ANC did not suppress antigen‐unspecific CD4+ T‐cell proliferation. This corresponds to results from He et al., who showed that NNC had suppressive activity on antigen‐specific proliferation of CD8^+^ T cells, while neutrophilic cells isolated from postpartum mice did not [22].

NNC depletion resulted in reduced survival after sepsis induction with both low (10,000 CFU/pup) or high dose (30,000 CFU/pup) inoculation of *E. coli* compared to control animals. This was accompanied by increased bacterial load in some organs mainly after low dose infection, hinting towards a loss of anti‐bacterial capacity by NNC depletion. This is in line with clinical conditions characterized by diminished granulopoesis, such as after bone marrow transplantation, or during functional defects of granulocytes, such as septic granulomatosis or other hereditary disorders, with high risk of bacterial infections [48, 49, 50], and with sepsis models in adult mice, where sepsis course deteriorated with increased bacterial load and low inflammation after neutrophil depletion [51, 52, 53]. Interestingly, bacterial load was only marginally higher in mice who died compared to mice who survived during the observation period of 48 h, which argues against a direct relationship between bacterial load and mortality.

Interestingly, in our neonatal model, pro‐inflammatory signals were increased after NNC depletion. This underscores, that NNC exhibit anti‐inflammatory as known for GR‐MDSC such as suppression of monocytes [16, 17] or T‐cell and NK‐cells function [10, 25]. The role MDSC during adult sepsis has been investigated in several studies in mice [54, 55, 56, 57, 58, 59] and humans [60, 61, 62, 63, 64, 65] but with controversial results. Some studies showed an improvement in survival by MDSC [54, 56, 58, 60] whereas others reported a deterioration of sepsis [61, 62, 63]. This may be due to the heterogeneous phenotype and the dynamic nature of MDSC in different settings [23, 24, 66]. The dynamic nature is emphasized by the work of Brudecki et al. who transferred MDSC from mice with polymicrobial sepsis either in the early stage or in the late stage of the sepsis to other mice also with polymicrobial sepsis. MDSC from the early stage of the sepsis increased proinflammatory cytokine production and early mortality, while MDSC from the late stage of sepsis suppressed inflammation and improved survival [56]. To our knowledge, the role of GR‐MDSC for neonatal sepsis has not yet been investigated in depth. Deshmukh et al. depleted neutrophilic cells by an anti‐Ly6G antibody and also observed an increased susceptibility to *E. coli* sepsis [21]. However, they did not analyze the functionality of the neutrophilic cells. Consequently, no statement can be made, whether these cells were anti‐ or pro‐inflammatory. We now complement these earlier data by adding that, in addition to their role in bacterial clearance, NNC also appear to dampen inflammation during neonatal sepsis, potentially improving disease outcome [67, 68, 69].

Antibody mediated Ly6G‐depletion, immediately after birth, lasted for at least one week in contrast to results in adult mice models, where depleting effect lasted only for up to 4 days [15, 70]. This implies that not only very early “fetal” NNC were depleted as intended, but presumably also the de novo generated mature, pro‐inflammatory neutrophils derived from the bone marrow. This might at least in part explain the harmful effects of NNC‐depletion during neonatal sepsis, potentially explaining the increased bacterial load in NNC‐depleted animals.

A limitation of our study is that fluorochrome‐conjugated anti‐Ly6G antibodies would not detect all circulating neutrophilic cells bound to the depleting anti‐Ly6G antibody. However, to the best of our knowledge, the technique we used in our experiments is the standard method for depletion of neutrophilic cells and has been used in several other studies in newborn mice [21, 71, 72]. A very elegantly conducted and recently published study has now shown that the use of anti‐Ly6G antibody in newborn mice leads to only a 50% reduction in neutrophilic cells. This was determined by performing intracellular staining in addition to extracellular staining and detecting neutrophilic cells that had bound the depleting antibody using an anti‐rat IgG2a antibody [73], suggesting that the effects we observe are less pronounced when NNC are completely absent. However, it is also possible that certain subpopulations of NNC are more or less efficiently depleted by anti‐Ly6G. Further studies are therefore needed to more comprehensively elucidate the role of NNC in neonatal sepsis. Another limitation is that due to the small sample sizes that can be obtained from newborn mice, we could only measure selected serum cytokines. Although an increase was observed in IL‐6 and MCP‐1 following Ly6G depletion, it should be noted that this does not capture the full complexity of the cytokine network. Moreover, it must be critically noted that, apart from the suppression of T‐cell proliferation by NNC, no further experiments were performed to comprehensively characterize immunosuppressive properties of NNC. Future studies involving functional and/or transcriptional analyses of immune cells from septic mice with and without NNC depletion would provide a better understanding of the immunoregulatory properties of NNC. The last limitation to be mentioned is that we did not analyze the influence of sex in our experiments although there is evidence that sex plays a role for adult murine sepsis [74]. To our knowledge, the impact of sex on neonatal sepsis has not yet been described and should be considered in future studies.

Interestingly, there were no major changes in the immune cell profile following NNC depletion. Given the increased mortality after NNC depletion, immune cell alterations would have been expected. Functional immunological analyses, especially analyses of T‐cell functions such as cytokine production and expression of exhaustion markers would be helpful to describe the immunological effects of NNC‐depletion during sepsis more comprehensively.

In conclusion, we show here that the depletion of NNC early in life fosters inflammation and increases mortality in neonatal sepsis. Our results contribute to a better understanding of the opposing characteristics of NNC and confirm previous work showing that suppressive neutrophils in the newborn are crucial for early inflammatory control [22]. The difficulty in differentiating these immunosuppressive cells from pro‐inflammatory mature neutrophils makes cell‐based approaches to prevent hyperinflammation in the newborn currently challenging. Further research is needed to better define this particular cell population and its functional property.

## Author Contributions

All authors were involved in drafting the article or revising it critically for important intellectual content, and all authors approved the final version to be published. J.S., S.D., T.L., J.H., S.D, J.R. C.G., and N.K.‐G. contributed to the acquisition of data, analysis, and interpretation of data. C.F.P. and T.O. provided critical feedback on intellectual content. J.S., N.K.‐G., and C.G. conceived the study and wrote the paper.

## Funding

This work was supported by research grants of the Ministerium für Wissenschaft, Forschung und Kunst Baden‐Württemberg and the European Social Fund Plus, the Deutsche Forschungsgemeinschaft (DFG) and the German Center for Infection Research (DZIF).

## Ethics Statement

No studies were performed on human samples. All experimental animal procedures were conducted according to German federal and state regulations (approval number K07/18).

## Conflicts of Interest

The authors declare no conflicts of interest.

## Supporting information




**Supporting File**: eji70171‐sup‐0001‐SuppMat.pdf.

## Data Availability

The datasets generated during and/or analyzed during the current study are available from the corresponding author upon reasonable requests.
