# Supplementary material for: Depletion of Neonatal Neutrophilic Cells Worsens the Outcome of E. coli Sepsis in Newborn Mice
Source: Eur J Immunol. 2026 Mar 29;56(4):e70171. doi: 10.1002/eji.70171 (PMC13033951; doi:10.1002/eji.70171)
Supplement: Supplementary file 1 — Supporting File: eji70171‐sup‐0001‐SuppMat.pdf. [file EJI-56-e70171-s001.pdf]

Supplementary Figure 1

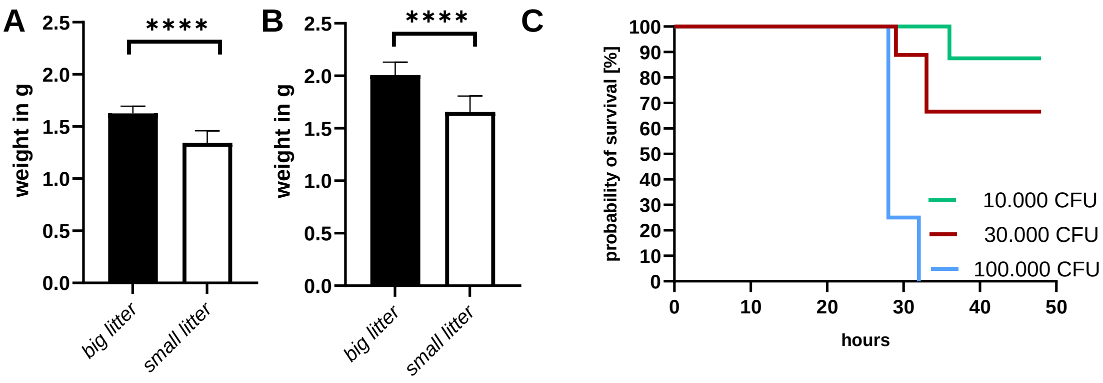

Supplementary Figure 1: Weight differences of pups from litters of different sizes and summary of survival rates after different doses of *E. coli* in mice at P2 in small litters.

(A, B) Sepsis was induced in newborn mice at the second day after birth (P2) by subcutaneous injection of 10.000/mouse CFU (A) or 30.000 CFU/mouse (B) *E.coli*. The litter size was either 5 pups or 9 pups. Bar graphs show the weight of animals born in small (A: n=16; B: n=17) or big litters (A: n=8; B: n=9) . p****<0,0001; Mann-Whitney test; mean and standard deviation are indicated. (C) Summary of mortalities after different doses of *E. coli* in mice infected at P2 in small litters. 10.000 CFU: n=8, 30.000 CFU: n=6, 100.000 CFU: n=4. Each n is representing a single mouse. Shown are mice from three litters (A-C).

Supplementary Figure 2

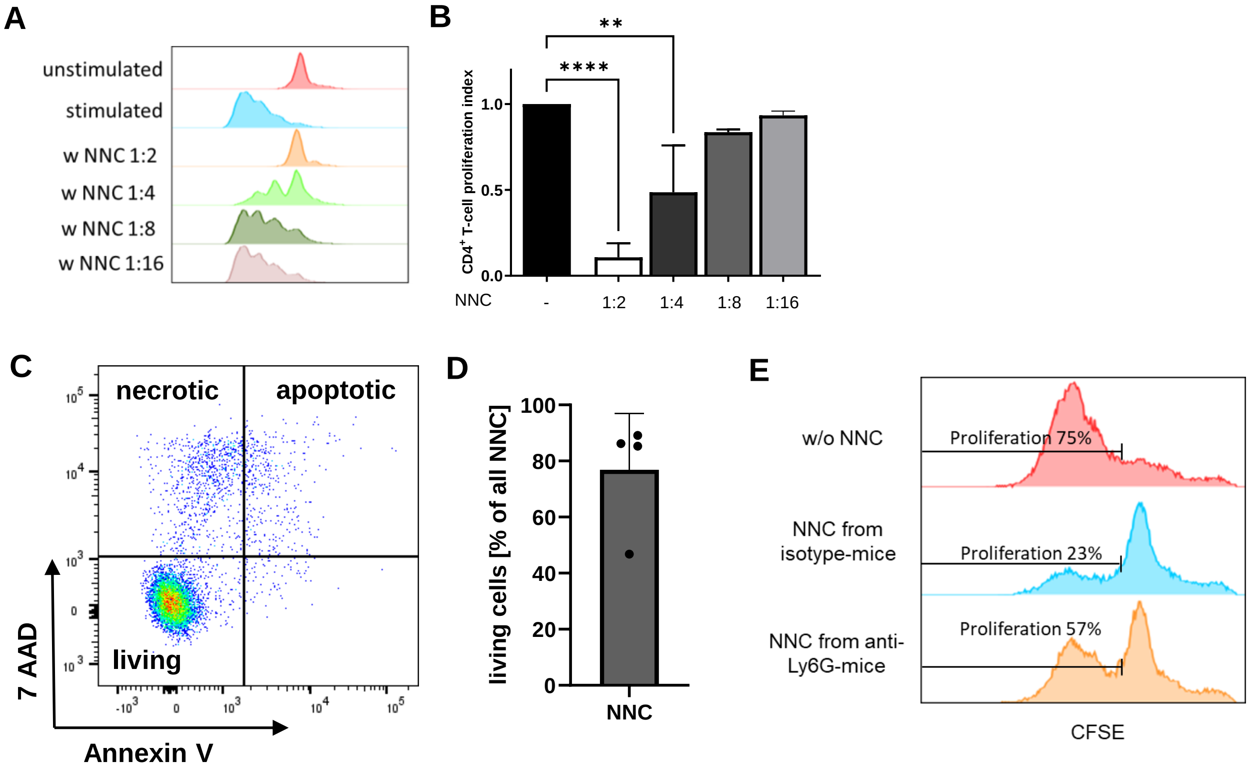

Supplementary Figure 2: Suppressive capacitiy and viability of NNC

(A, B) Newborn mice at P1 were euthanized and spleens were collected. NNC were isolated by MACS and added in different ratios to $CD4^+$ T-cells, freshly isolated from spleens of adult mice, stained with CFSE and stimulated with anti-CD3/CD28 microbeads. After four days, proliferation of $CD4^+$ T-cells was assessed by CFSE dye dilution. The proliferation index was determined as ratio of T-cell proliferation with and without addition of NNC. (A) Representative histogram plots showing proliferation of unstimulated $CD4^+$ T-cells (red histogram), stimulated $CD4^+$ T-cells without (blue histogram) and with the addition of NNC in a 1:2 ratio (orange histogram), a 1:4 ratio (green histogram), a 1:8 ratio (olive histogram) and a 1:16 ratio (rose histogram). (B) Bar graph showing the inhibitory effect of NNC in different ratios on $CD4^+$ T-cell proliferation by CFSE dye dilution. Proliferation of $CD4^+$ T-cells after stimulation and without addition of NNC was set to 1. Bars show mean and standard deviation of 8 samples from 8 independent experiments; $^{**}p < 0.01$, $p^{****}<0.0001$; Kruskal-Wallis test.

(C+D) Newborn mice at P1 were euthanized and spleens were collected. NNC were isolated by MACS and cultured for 4 days. Viability the NNC was measured using a AnnexinV/7-AAD Apoptosis Detection Kit. (C) Representative density plot shows living cells (lower left quadrant, living), necrotic cells (upper left quadrant, necrotic) and apoptotic cells (upper right quadrant, apoptotic). (D) Bar graph shows percentages of living cells of NNC. Bar shows mean and standard deviation from 4 independent experiments. Each dot (=n) is representing a single mouse. Mice for the experiments came from two litters.

(E) Newborn mice received either an intraperitoneal injection of an anti-Ly6G antibody (anti Ly6G-mice) to deplete NNC or an isotype control (isotype-mice) on P1. On P2 mice were euthanized and spleens were collected. NNC were isolated by (MACS) and added in a ratio of 1:4 to $CD4^+$ T-cells freshly isolated from spleens of adult mice, stained with CFSE and stimulated with anti-CD3/CD28 microbeads. After four days, proliferation of $CD4^+$ T-cells was assessed by CFSE dye dilution. Representative histogram plots showing proliferation of stimulated $CD4^+$ T-cells without (red histogram, positive control; w/o NNC) and with addition of NNC after injection of an isotype control (blue histogram, NNC from isotype-mice) or after injection of an anti-Ly6G antibody (orange histogram, NNC from anti-Ly6G-mice).

Supplementary Figure 3

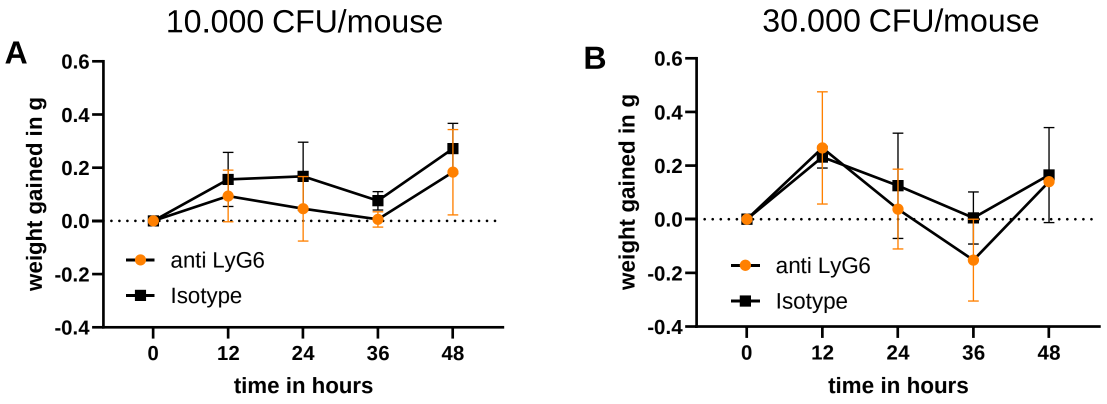

Supplementary Fig. 3: Weight gain after sepsis induction with and without NNC-depletion.

Newborn mice received either an intraperitoneal injection of an anti-Ly6G antibody (anti Ly6G, orange dot) to deplete NNC or an isotype control antibody (Isotype, black dots) at the first day of life (P1). At P2 mice were injected subcutaneously with either 10.000 CFU (A) or 30.000 CFU *E.coli* (B). The first weight was measured at the timepoint of sepsis induction. The pups were weighted every 12 hours. Line charts show weight gain of animals after sepsis induction with 10.000 CFU *E. coli* (A; anti Ly6G: n=5, isotype: n=5) or 30.000 CFU *E. coli* (B; anti Ly6G: n=7, isotype: n=8). Dots show mean and standard deviation. Each n is representing a single mouse. Mice for the experiments came from three litters.

Supplementary Figure 4

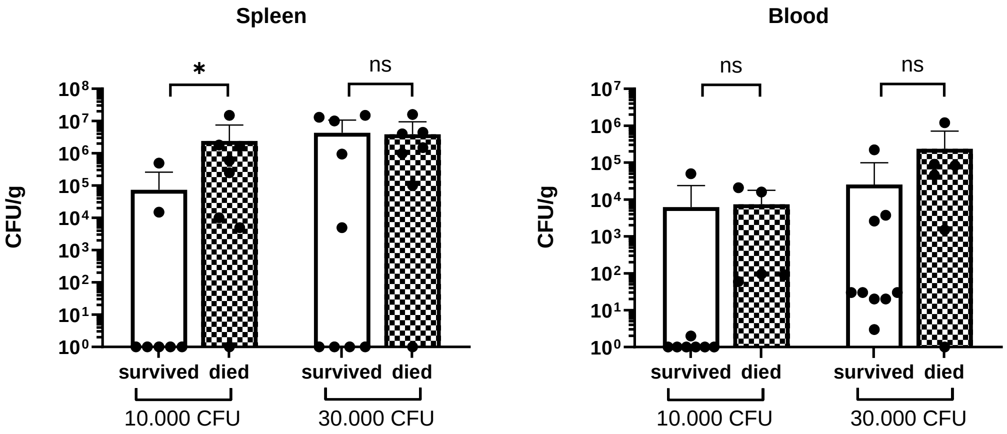

Supplementary Figure 4: Correlation of bacterial load after induction neonatal E.coli sepsis after the survival

Newborn mice received either an intraperitoneal injection of an anti-Ly6G antibody (anti Ly6G) to deplete NNC or an isotype control antibody (Iso) at the first day of life (P1). At P2 mice were injected subcutaneously with either 10.000 CFU or 30.000 CFU E. coli. Injection of PBS served as negative control. The pups were sacrificed when reaching the criteria for critical illness or 48h after sepsis induction. Spleens and blood were collected. Organs were homogenized and samples were diluted with PBS. Suspensions were incubated for 24 hours on Columbia agar plates with 5% sheep blood and colony forming units (CFU) were counted to calculate CFU per gram (g) organ weight or per milliliter (ml) fluid (blood). Bars showing CFUs per gram organ weight (A) or per ml (B) in spleens and blood (B) of mice who survived (survived, white bars) or mice of died (died, striped bars) with sepsis induction with 10.000 CFU (left side) or 30.000 CFU (right side). Bars show mean and standard deviation. n= 5-9, *<p:0,05, ns: not significant, unpaired t test. Each dot (=n) is representing a single mouse and experiment. Mice for the experiments came from three litters.

Supplementary Figure 5

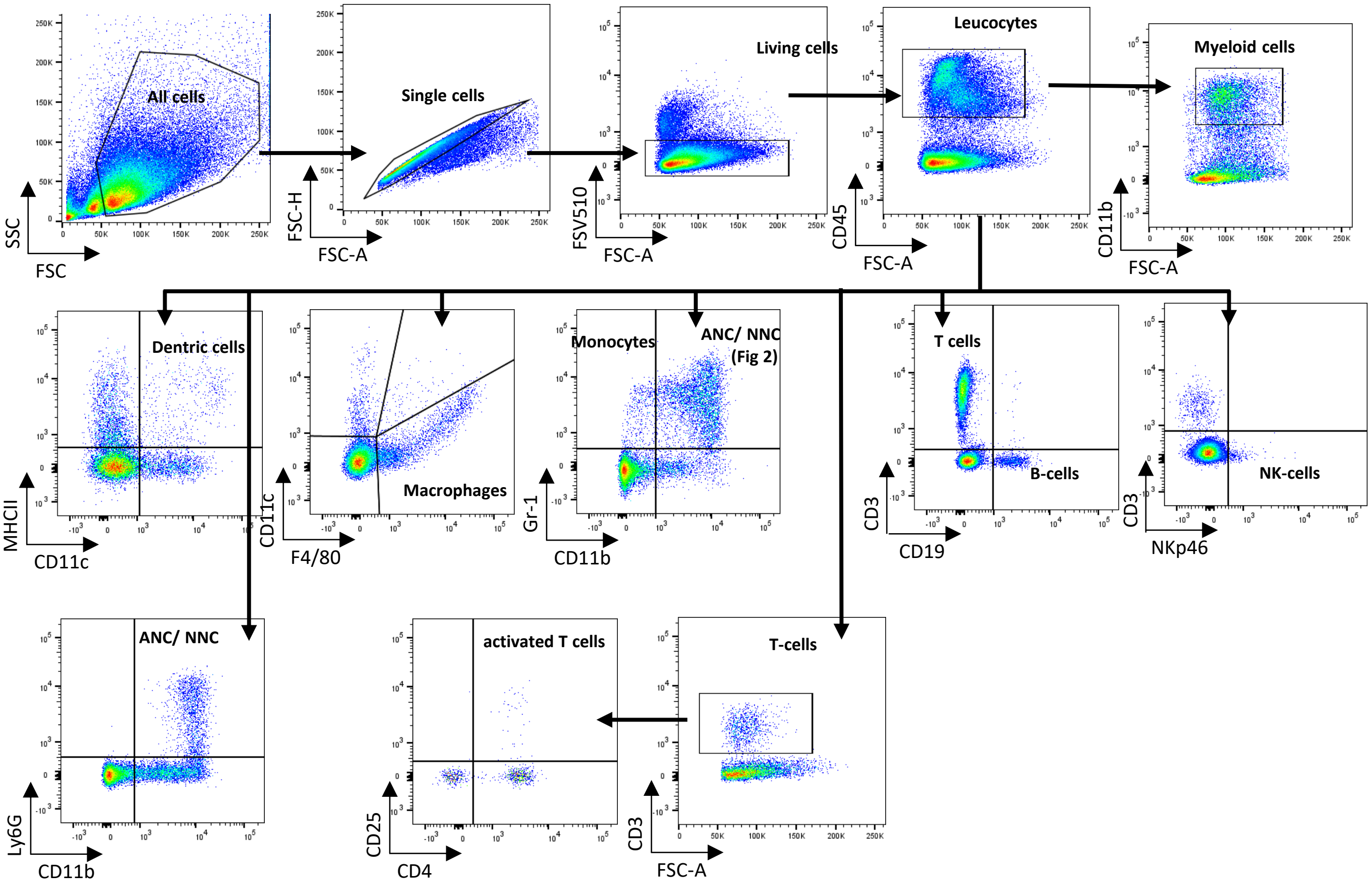

Supplementary Figure 5: Gating strategy for immune cell populations in spleens and livers. Representative density plots show the gating strategy for immune cell populations in spleens and livers. Debris was excluded by gating on cells in SSC/FSC. Doublets were excluded and cells were pre-gated on living cells and leukocytes. Within CD45$^+$ leukocytes, immune cell subsets were defined as follows:T-cells by CD3$^+$, activated T cells by CD3$^+$/CD4$^+$/CD25$^+$, B-cells by CD3$^-$/CD19$^+$, NK-cells by CD3$^-$/NKp46$^+$, neutrophilic cells by CD11b$^+$/Gr-1$^+$, neonatal or adult neutrophilic cells (NNC or ANC) by CD11b$^+$/ Ly-6G $^+$, dendritic cells by CD11c$^+$/MHCII$^+$, macrophages by CD11c$^-$/F4/80$^+$ and monocytes by CD11b$^+$/Gr-1$^-$.

Supplementary Figure 6

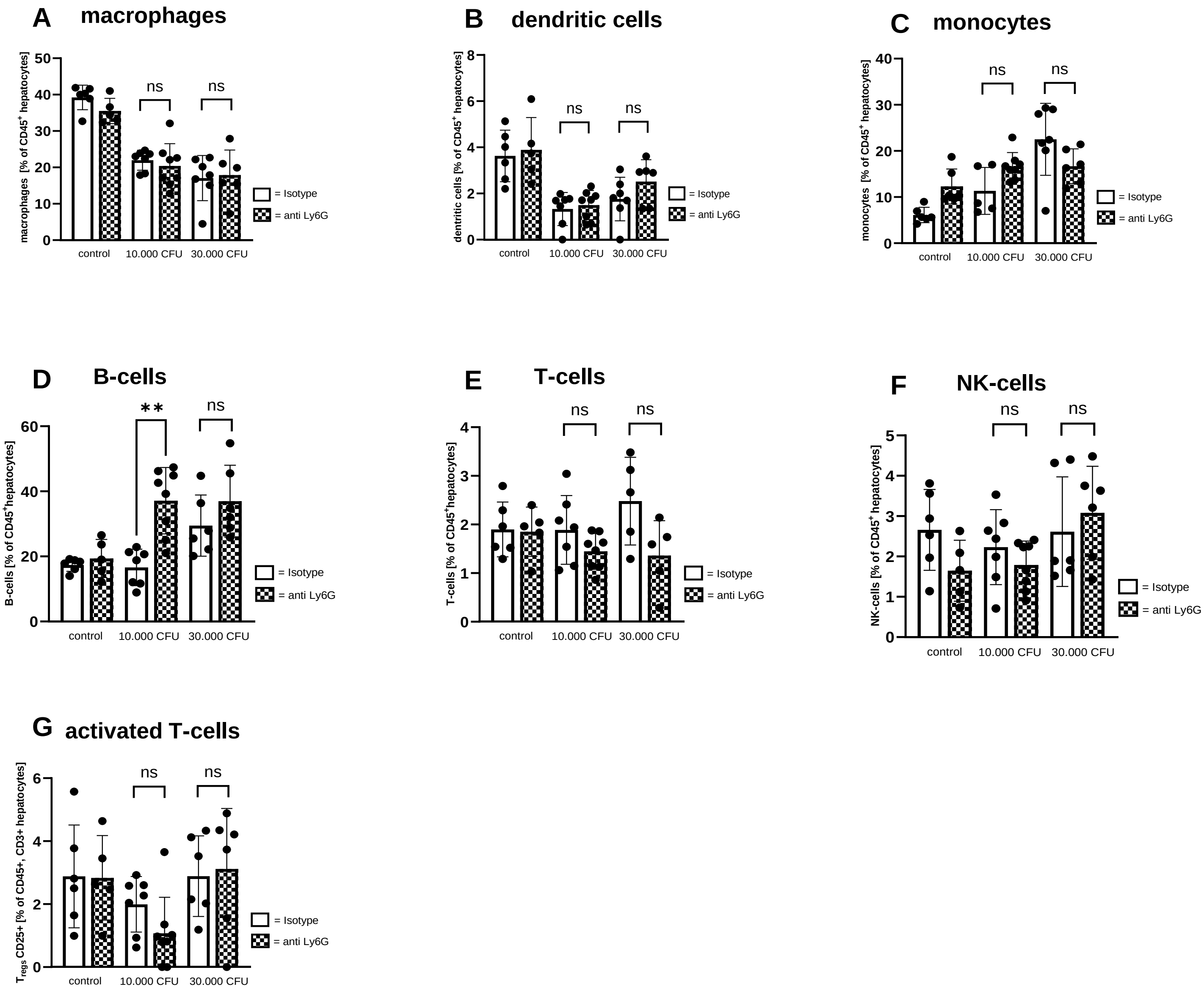

Supplementary Figure 6: Immune cell populations in livers after depletion of Ly6G-positive NNC

Newborn mice received either an intraperitoneal injection of an anti-Ly6G antibody (anti Ly6G) to deplete NNC or an isotype control antibody (Isotype) at (P1). At P2 mice were injected subcutaneously with either 10.000 CFU or 30.000 CFU E. coli. Injection of PBS served as negative control. The pups were sacrificed when reaching the criteria for critical illness or 48h after sepsis induction. Livers were collected and homogenized to obtain single cell suspensions. Immune cell subsets were analyzed by flow cytometry. Scatter diagrams with bars showing percentages of macrophages (A), dendritic cells (B), monocytes (C), B-cells (D), T-cells (E), and NK-cells (F) and activated T-cells of leukocytes of control mice (isotype, white bars) or NNC-depleted mice (anti-Ly6G, striped bars) without sepsis induction (control) or with sepsis induction with 10.000 CFU or 30.000 CFU. n=6-7, unpaired t test. Each dot (=n) is representing a single mouse. Mice for the experiments came from three litters.
